# Effects of black soldier fly (*Hermetia illucens*) larvae meal on production performance, egg quality, and physiological properties in laying hens: A meta-analysis

**DOI:** 10.14202/vetworld.2024.1904-1913

**Published:** 2024-08-24

**Authors:** Faisal Fikri, Agus Purnomo, Shekhar Chhetri, Muhammad Thohawi Elziyad Purnama, Hakan Çalışkan

**Affiliations:** 1 Division of Veterinary Medicine, Department of Health and Life Sciences, Faculty of Health, Medicine, and Life Sciences, Universitas Airlangga, Banyuwangi, Indonesia; 2 Department of Veterinary Surgery and Radiology, Faculty of Veterinary Medicine, Universitas Gadjah Mada, Yogyakarta, Indonesia; 3 Department of Animal Science, College of Natural Resources, Royal University of Bhutan, Lobesa, Punakha, Bhutan; 4 Department of Biology, Graduate School of Natural and Applied Sciences, Eskişehir Osmangazi Üniversitesi, Eskişehir, Türkiye; 5 Department of Biology, Faculty of Science, Eskişehir Osmangazi Üniversitesi, Eskişehir, Türkiye

**Keywords:** black soldier fly, egg quality, food production, laying hen, meta-analysis

## Abstract

**Background and Aim:**

The primary components of fat and protein in chicken diets are fishmeal and soybean; however, due to limited supply and high costs, several efforts have been made to utilize alternative feedstuffs. The potential of black soldier fly larvae (BSFL) as a substitute for fat and protein has been extensively studied, but the findings are not consistent. This study used a meta-analysis approach to investigate the integrated efficacy of BSFL supplementation on laying hen production performance, egg quality, and physiological properties.

**Materials and Methods:**

The articles were retrieved from PubMed, Scopus, ScienceDirect, Cochrane Library, and ProQuest. The retrieved references were examined for potential inclusion. The relevant findings of the included studies were then extracted. Fixed-effects, standard mean difference, 95% confidence intervals, and heterogeneity models were analyzed using the Review Manager website version (Cochrane Collaboration, UK).

**Results:**

A total of 24 papers from 17 different nations across five continents have been selected for meta-analysis out of the 3621 articles that were reviewed. The current meta-analysis demonstrated that providing BSFL meals significantly favored feed efficiency, haugh units, albumen quality, eggshell quality, serum glucose, and lipid levels. In addition, significant trends in alanine transaminase, alkaline phosphatase, magnesium, phosphorus, chlorine, and iron levels were observed in blood urea nitrogen, uric acid, creatinine, lactate dehydrogenase, creatine kinase, glutathione peroxidase, and malondialdehyde. On the other hand, it was revealed that there was no favorable effect on weight gain, laying, yolk quality, and hematological profile.

**Conclusion:**

The meta-analysis confirmed that BSFL meals can be utilized to optimize feed efficiency, haugh units, albumen, eggshell quality, liver, renal, and cellular physiology of laying hens, although they did not significantly increase body weight gain, laying production, and hematological profiles.

## Introduction

Egg production is predicted to rise at a rate of more than 50% over the next 20 years, making it the second-fastest-growing sector globally. Within the European Union (EU), the amount of laying hens exceeds 350 million. Annually, laying hens yield approximately 6.7 million tons of eggs [1]. In addition to marketing requirements, the EU occasionally implements other market assistance initiatives to assist egg farmers. Egg products, particularly in liquid form, pulverized, whole eggs or their separated primary components, as well as yolk and albumen, are highly demanded by the industry, despite the fact that eggs are a critical source of protein molecules, essential amino acids, lipids, and various trace elements [2]. However, the industry that raises laying hens is influenced by several significant variables, such as the availability of feed, which has prompted the rise in consumer demand for hen products, including eggs [3].

At present, fish meal and soybeans are the primary components of fat and protein in chicken diets. Fish meal and fish oil are produced by tiny benthic forage fish, which are becoming less common because of overfishing in the ocean. In addition, a finite amount of land is available for soy growth worldwide. This has stimulated research on alternative protein sources that are low-cost, have good nutrient content, and are environmentally sustainable [4]. In their natural environment, poultry eat insects, which are considered an essential source of protein [5]. In Europe, feeding live insects to chickens is already permitted. This includes larvae of the black soldier fly (BSFL) (*Hermetia illucens*). Due to their exceptional capacity to consume various organic wastes and high protein content (up to 39%–64%), BSFLs have been used in insect-based meals [6].

Several recent studies have investigated the effectiveness of BSFL feeding on the characteristics of growth performance, egg quality, hematological traits, serum proteins, glucose, lipids, electrolytes, liver function, and renal function in laying hens [7–30]. However, no systematic study that incorporates data from multiple studies on the effectiveness of BSFL in laying hens has been reported using a meta-analysis methodology. It is crucial that a meta-analysis study be conducted to elucidate and evaluate data that can present inconsistent outcomes from earlier investigations. Thus, we evaluated the effects of BSFL-based diets on the production performance, egg quality, and physiological properties of laying hens using a meta-analysis approach.

## Materials and Methods

### Ethical approval

The study followed the preferred reporting items for systematic reviews and meta-analysis (PRISMA) guidelines [31]. Ethics approval was not required because this study did not involve any animals.

### Study period and location

The screening procedure for relevant literature, data compilation, and data analysis was performed from December 2023 to March 2024 at the Department of Biology, Faculty of Science, Eskişehir Osmangazi Üniversitesi, Eskişehir, Türkiye.

### Search strategy and study selection

Articles from PubMed, Scopus, Science Direct, Cochrane Library, and ProQuest were individually screened by each author to identify pertinent studies that discussed the effectiveness of BSFL in laying hens. The PICO method (Table-1) was implemented to formulate the study questions (P, population = Laying hens; I, intervention = Black soldier fly; C, comparison = Control; and O, outcomes = Egg quality and growth performance). The most relevant search terms were “black soldier fly, *H. illucens*, laying hens, egg quality, and growth performance.” The search for each database was restricted to English rather than the date. MeSH terms contained all relevant and comprehensive keywords. The databases’ sample search algorithm was as follows: #1 chicken [MeSH Terms] OR hens [Title/Abstract] OR layer [Title/Abstract] OR laying hens [Title/Abstract] #2 Black Soldier Fly [MeSH Terms] OR *Hermetia illucens* [Title/Abstract] OR maggot [Title/Abstract] #3 egg quality [MeSH Terms] AND production performance [MeSH Terms] OR growth performance [Title/Abstract].

**Table-1:** Searching strategy based on the PICO methods.

PICO items	PICO	Keywords
Problems, patients, population	Laying hens	Chicken [MeSH terms] OR hens [title/abstract] OR layer [title/abstract] OR laying hens [title/abstract]
Intervention	Black soldier fly	Black soldier fly [MeSH terms] OR *Hermetia illucens* [title/abstract] OR maggot [title/abstract]
Comparison, control	Control	“Control groups” [MeSH terms]
Outcomes	Primary outcome: Egg quality, production performance Secondary outcomes: Growth performance	Egg quality [MeSH Terms] AND production performance [MeSH Terms] OR growth performance [title/abstract]

PICO=Population, intervention, comparison, and outcome

### Eligibility criteria

**Figure-1:**
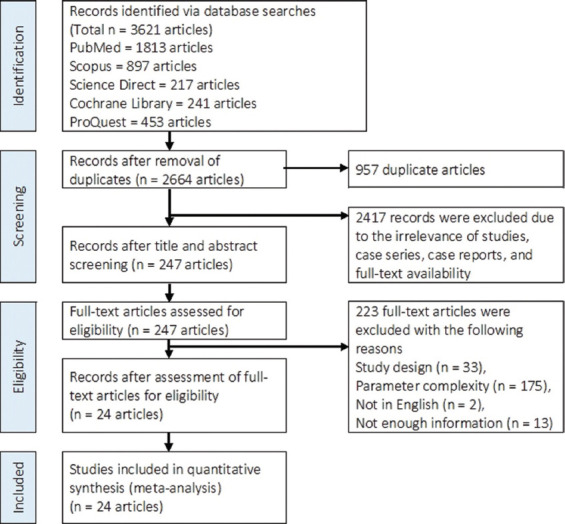
Preferred reporting items for systematic reviews and meta-analysis flow diagram of the study selection process.

Listed below eligibility criteria were used in the selection of the articles: (a) English articles; (b) original research articles; (c) full-text available and open access articles; (d) *in vivo* studies; (e) randomized clinical trials; (f) BSFL meals were used as the feed; and (g) parameters were disclosed in detail. The criteria for exclusion were as follows: (1) duplicated studies; (2) irrelevance of studies; (3) unavailable full-text; (4) irrelevant study design (case series, case report); (5) not available in English; and (6) not enough information. The PRISMA technique was adopted for the identification, screening, eligibility, and inclusion of articles during the article selection process (Figure-1).

### Data extraction

The authors extracted and checked the information from the included studies. The extracted data’s characteristics were presented as follows: (1) study reference; (2) study year; (3) country; (4) laying hen breed/hybrid; (5) BSFL ratio; (6) laying hen age; and (7) sample size.

Detailed data on the outcomes were also extracted, including the production performance (initial and final body weight, weight gain, feed intake, feed efficiency, and laying frequency); egg quality (egg weight, egg mass, haugh unit, egg yolk, egg albumen, and eggshell quality); hematological characteristics; serum proteins, glucose, and lipids; serum liver enzymes; electrolyte levels; renal physiology; and plasma antioxidant capacity.

### Statistical analysis

The online version of Review Manager (Cochrane Collaboration, UK) was used for the statistical analysis. To compute the standard mean difference (SMD), fixed effect, and 95% confidence intervals, a pairwise meta-analysis of the results was performed between the BSFL and control groups. Chi-square test (χ^2^) was used to assess the heterogeneity between the studies. When p < 0.05 and the I^2^ value >50%, significant heterogeneity was deemed to exist. The data visualized in the forest plots were validated and represented in the tables.

## Results

### Identification and selection of studies

Five electronic databases yielded 3,621 articles (PubMed had 1,813 items, Scopus had 897 articles, Science Direct had 217 articles, Cochrane Library had 241 articles, and ProQuest had 453 articles). Of these, 957 were duplicates, and 2,417 were omitted because full-text versions of the studies, case reports, and case series were no longer relevant. After screening the titles and abstracts, 247 full-text publications remained. Among them, 223 studies were deemed ineligible for inclusion in the meta-analysis due to factors such as parameter complexity, improper study design, non-English language, and insufficient data. The meta-analysis ultimately included 24 articles that were eligible for inclusion. The flow chart depicts the process of selecting studies (Figure-1).

### Characteristics of the included studies

A total of 4,549 samples from 24 included studies representing different treatment ratios of BSFL diets were analyzed. Data were published from 17 different countries across five continents (eight from Europe, four from North America, one from Africa, one from Australia, and 10 studies from Asia). Of the 4,549 laying hen samples evaluated, 54 were Arabic breed, 192 Babcock White, 96 Bovans Brown, 360 Bovans Robust White, 144 Charoen Pokphand Brown, 44 Dekalb White, 1,782 Hy-Line Brown, 570 ISA Brown, 87 Julia, 266 Lohmann Brown Classic, 90 Novogen Brown, 216 Shaver white leghorns, 216 white leghorn, and 432 Xuefeng black-bone (Table-2) [7–30].

**Table-2:** Characteristics of the studies.

Studies	Study year	Country	Laying hen breeds	Black soldier fly larvae ratio (%)	Age of laying hens (weeks)	Samples (n)
Al-Qazzaz *et al*. [7]	2016	Malaysia	Arabic	0; 5; and 1	36–48	54
Aslam [8]	2023	Sweden	Bovans robust white	0; 21.8; and 59.5	30–49	360
Bejaei and Cheng [9]	2020	Canada	Novogen brown	0; 10; and 18	20–35	90
Bovera *et al*. [10]	2018	Italy	Hy-line brown	0; 25; and 50	20–40	162
Heuel *et al*. [11]	2021	Switzerland	Lohmann brown classic	0; and 20	28–35	50
Irawan *et al*. [12]	2019	Indonesia	ISA brown	0; and 8	18–26	200
Kim *et al*. [13]	2022	Korea	Hy-Line brown	0; 50; and 100	26–33	144
Liu *et al*. [14]	2021	China	Xuefeng black-bone	0; 1; 3; and 5	45–50	432
Lokaewmanee *et al*. [15]	2023	Thailand	Charoen pokphand brown	0; 10; 20; and 30	25–37	144
Marono *et al*. [16]	2017	Italy	Lohmann brown classic	0; and 17	24–45	108
Mwaniki *et al*. [17]	2018	Canada	Shaver white leghorns	0; 5; and 7.5	19–27	108
Mwaniki *et al*. [18]	2020	Canada	Shaver white leghorns	0; 10; and 15	28–43	108
Nassar *et al*. [19]	2023	Saudi Arabia	Hy-Line brown	0; 3; 6; 9; and 12	40–50	270
Navasero *et al*. [20]	2022	Philippines	Babcock white	0; 1.5; and 3	54–62	192
Park *et al*. [21]	2021	Korea	Hy-line brown	0; 2; and 4	26–33	144
Patterson *et al*. [22]	2021	USA	White leghorn	0; 8; 16; and 24	51–55	216
Ruhnke *et al*. [23]	2018	Australia	ISA brown	0; and 90	50–62	160
Secci *et al*. [24]	2018	Italy	Lohmann brown classic	0; and 17	24–45	108
Secci *et al*. [25]	2020	Italy	Hy-line brown	0; 25; and 50	20–35	162
Star *et al*. [26]	2020	Netherlands	Dekalb white	0; and 10	67–78	44
Wamai *et al*. [27]	2024	Kenya	ISA brown	0; 25; 50; 75; and 100	18–60	210
Yan *et al*. [28]	2023	China	Hy-line brown	0; 1; 2; and 3	60–68	900
Zawisza *et al*. [29]	2023	Poland	Bovans brown	0; 5; 10; and 15	25–36	96
Zhao *et al*. [30]	2022	Japan	Julia	0; 1.5; and 3	25–52	87

### Growth performance

According to the present meta-analysis, feeding BSFL meals had a favorable effect on feed efficiency (SMD = 0.53; p < 0.00001). However, feeding BSFL meals had a negative correlation with weight gain (SMD = −0.27; p = 0.001), feed intake (SMD = −0.12; p = 0.02), or laying production (SMD = −0.88; p < 0.00001) (Table-3).

**Table-3:** Effects of black soldier fly larvae on laying hen production.

Parameter	n	Fixed effect			Heterogeneity		
	
		SMD	95% CI	p-value	χ^2^	I^2^(%)	p-value
Initial body weight (kg)	928	−0.41	−0.54–−0.28	<0.00001	96.13	91	<0.00001
Final body weight (kg)	1128	−0.42	−0.55–−0.29	<0.00001	463.22	97	<0.00001
Weight gain (g)	1202	−0.27	−0.43–−0.11	0.001	1177.04	99	<0.00001
Feed intake (g/hen/day)	1964	−0.12	−0.23–−0.02	0.02	1143.18	99	<0.00001
Feed efficiency (g feed/g egg)	2056	0.53	0.42–0.64	<0.00001	1216.33	99	<0.00001
Laying (%)	1912	−0.88	−1.01–−0.76	<0.00001	1451.46	99	<0.00001

n=Total sample, SMD=Standard mean difference, 95% CI=95% confidence interval, I^2^ =Primary index for reporting heterogeneity

### Egg quality

The evaluation of egg quality revealed that feeding BSFL had a favorable effect on haugh units (SMD = 0.41; p < 0.00001), albumen height (SMD = 0.47; p < 0.00001), shell thickness (SMD = 0.29; p < 0.0001), and shell weight (SMD = 1.01; p < 0.00001). Meanwhile, no significant differences were observed for egg mass (SMD = −0.04; p = 0.61), yolk weight (SMD = −0.02; p = 0.88), yolk height (SMD = −0.39; p = 0.11), albumen weight (SMD = 0.08; p = 0.46), and eggshell weight (SMD = −0.12; p = 0.30) (Table-4).

**Table-4:** Effects of black soldier fly larvae on egg quality in laying hens.

Parameter	n	Fixed effect			Heterogeneity		
	
		SMD	95% CI	p-value	χ^2^	I^2^ (%)	p-value
Egg weight (g)	2480	−0.11	−0.20–−0.03	0.01	865.09	97	<0.00001
Egg mass (g/day)	860	−0.04	−0.21–0.12	0.61	553.18	98	<0.00001
Haugh unit	1462	0.41	0.29–0.53	<0.00001	664.88	98	<0.00001
Yolk (g)	506	−0.20	−0.39–−0.00	0.05	184.02	97	<0.00001
Yolk color	1362	−0.60	−0.75–−0.44	<0.00001	1320.80	99	<0.00001
Yolk height (mm)	78	−0.39	−0.87–0.08	0.11	15.16	93	<0.00001
Yolk (% weight)	388	−0.02	−0.23–0.20	0.88	114.54	97	<0.00001
Yolk index	388	−0.79	−1.03–−0.55	<0.00001	173.50	99	<0.00001
Albumen (g)	506	−0.14	−0.33–0.05	0.15	134.52	96	<0.00001
Albumin height (mm)	632	0.47	0.29–0.64	<0.00001	245.33	98	<0.00001
Albumin (% weight)	448	0.08	−0.13–0.29	0.46	206.08	98	<0.00001
Albumen index	172	−0.91	−1.35–−0.47	<0.0001	161.93	99	<0.00001
Ratio of yolk to albumin	506	−0.69	−0.88–−0.51	<0.00001	56.40	89	<0.00001
Shell breaking strength (N)	1014	−0.25	−0.40–−0.09	0.002	714.63	99	<0.00001
Shell thickness (mm)	1322	0.29	0.15–0.43	<0.0001	1026.54	99	<0.00001
Shell weight (g)	918	1.01	0.85–1.16	<0.00001	411.22	98	<0.00001
Shell (% weight)	388	−0.12	−0.35–0.11	0.30	191.57	98	<0.00001

n=Total sample, SMD=Standard mean difference, 95% CI=95% confidence interval, I^2^ =Primary index for reporting heterogeneity

### Hematological and electrolyte profiles and liver and renal physiology

BSFL meals had no significant effect on any of the hematological trait parameters (Table-5). On the other hand, we revealed that BSFL meals possibly had a negative effect on the albumin-globulin ratio (SMD = 1.64; p < 0.00001), glucose (SMD = 0.47; p = 0.0006), cholesterol (SMD = 0.45; p = 0.0002), and triglycerides (SMD = 0.76; p < 0.0001) (Table-6).

**Table-5:** Effects of black soldier fly larvae on the hematological traits of laying hens.

Parameter	n	Fixed effect			Heterogeneity		
	
		SMD	95% CI	p-value	χ^2^	I^2^(%)	p-value
Hematocrit (%)	208	−0.73	−1.03–−0.42	<0.00001	56.46	98	<0.00001
Hemoglobin (g/dL)	208	−1.04	−1.33–−0.75	<0.00001	7.49	87	0.006
Erythrocytes (×10^6^/mm^3^)	208	−0.80	−1.10–−0.51	<0.00001	39.74	97	<0.00001
Leukocytes (×10^3^/mm^3^)	208	−0.29	−0.57–−0.02	0.04	2.96	66	0.09
Heterophils (%)	208	−0.75	−1.08–−0.43	<0.00001	103.19	99	<0.00001
Lymphocytes (%)	208	−0.70	−1.16–−0.24	0.003	251.43	100	<0.00001
Monocytes (%)	208	−1.07	−1.37–−0.77	<0.00001	19.57	95	<0.00001
Ratio (H/L)	208	−0.14	−0.41–0.13	0.32	0.57	0	0.45

n=Total sample, SMD=Standard mean difference, 95% CI=95% confidence interval, I^2^=Primary index for reporting heterogeneity

**Table-6:** Effects of black soldier fly larvae on serum protein, glucose, and lipid content in laying hens.

Parameter	n	Fixed effect			Heterogeneity		
	
		SMD	95% CI	p-value	χ^2^	I^2^(%)	p-value
Total protein (g/dL)	372	−1.18	−1.44–−0.92	<0.00001	191.85	98	<0.00001
Albumin (g/dL)	264	−0.66	−0.92–−0.41	<0.00001	27.88	93	<0.00001
Globulin (g/dL)	216	−1.65	−2.01–−1.29	<0.00001	102.98	99	<0.00001
Albumin/globulin ratio	216	1.64	1.28–1.99	<0.00001	99.16	99	<0.00001
Glucose (mg/dL)	216	0.47	0.20–0.74	0.0006	0.48	0	0.49
Cholesterol (mg/dL)	472	0.45	0.21–0.68	0.0002	364.76	99	<0.00001
Triglycerides (mg/dL)	372	0.76	0.41–1.12	<0.0001	461.22	99	<0.00001
HDL (mg/dL)	148	−0.77	−1.15–−0.38	<0.0001	64.17	98	<0.00001
LDL (mg/dL)	148	−0.24	−0.57–0.09	0.15	12.38	92	0.0004

n=Total sample, SMD=Standard mean difference, 95% CI=95% confidence interval, I^2^ =Primary index for reporting heterogeneity, HDL=High-density lipoprotein, LDL=Low-density lipoprotein

Meanwhile, this meta-analysis investigated the effects of black soldier larvae meal on several physiological parameters, including liver, renal, electrolyte, and antioxidant activities. As a result, we report favorable effects on the following parameters, that is, alanine transaminase (ALT) (SMD = 0.30; p = 0.03), alkaline phosphatase (ALP) (SMD = 0.31; p = 0.02) (Table-7), magnesium (Mg) (SMD = 0.75; p < 0.0001), phosphorus (P) (SMD = 0.67; p < 0.0001), chlorine (Cl) (SMD = 0.98; p < 0.00001), iron (Fe) (SMD = 0.14; p = 0.44) (Table-8), blood urea nitrogen (BUN) (SMD = 0.86; p < 0.00001), uric acid (SMD = 0.23; p = 0.09), creatinine (SMD = 1.37; p < 0.00001), lactate dehydrogenase (SMD = 0.43; p = 0.001), creatine kinase (SMD = 0.67; p = 0.0003) (Table-9), glutathione peroxidase (GSH-PX) (SMD = 1.39; p < 0.00001), and malondialdehyde (MDA) (SMD = 4.78; p < 0.00001) (Table-10).

**Table-7:** Effects of black soldier fly larvae on serum liver enzyme levels in laying hens.

Parameter	n	Fixed effect			Heterogeneity		
	
		SMD	95% CI	p-value	χ^2^	I^2^(%)	p-value
AST (U/L)	264	−0.35	−0.63–−0.06	0.02	137.73	99	<0.00001
ALT (U/L)	264	0.30	0.03–0.57	0.03	106.74	98	<0.00001
GGT (U/L)	216	−0.89	−1.19–−0.60	<0.00001	35.98	97	<0.00001
ALP (U/L)	264	0.31	0.05–0.58	0.02	69.18	97	<0.00001

n=Total sample, SMD=Standard mean difference, 95% CI=95% confidence interval, I^2^=Primary index for reporting heterogeneity, AST=Aspartate aminotransferase, ALT=Alanine transaminase, GGT=Gamma-glutamyl transferase, ALP=Alkaline phosphatase

**Table-8:** Effects of black soldier fly larvae on the reduction of electrolyte levels in laying hens.

Parameter	n	Fixed effect			Heterogeneity		
	
		SMD	95% CI	p-value	χ^2^	I^2^(%)	p-value
Mg (mg/dL)	264	0.75	0.40–1.09	<0.0001	242.00	99	<0.00001
P (mg/dL)	264	0.67	0.34–0.99	<0.0001	214.26	99	<0.00001
Ca (mg/dL)	372	−1.26	−1.53–−0.99	<0.00001	217.17	99	<0.00001
Cl (mmol/L)	216	0.98	0.69–1.27	<0.00001	12.71	92	0.0004
Fe (mcg/L)	264	0.14	−0.22–0.51	0.44	281.30	99	<0.00001

n=Total sample, SMD=Standard mean difference, 95% CI=95% confidence interval, I^2^=Primary index for reporting heterogeneity, Mg=Magnesium, *P=*Phosphorus, Ca=Calcium, Cl=Chlorine, Fe=Iron

**Table-9:** Effects of black soldier fly larvae on renal physiology in laying hens.

Parameter	n	Fixed effect			Heterogeneity		
	
		SMD	95% CI	p-value	χ^2^	I^2^(%)	p-value
BUN (mg/dL)	216	0.86	0.49–1.24	<0.00001	181.44	99	<0.00001
Uric acid (mg/dL)	264	0.23	−0.03–0.49	0.09	69.53	97	<0.00001
Creatinine (mg/dL)	216	1.37	0.96–1.78	<0.00001	197.91	99	<0.00001
Lactate dehydrogenase (U/L)	264	0.43	0.18–0.69	0.001	51.85	96	<0.00001
Creatine kinase (U/L)	216	0.67	0.31–1.04	0.0003	178.88	99	<0.00001

n=Total sample, SMD=Standard mean difference, 95% CI=95% confidence interval, I^2^=Primary index for reporting heterogeneity, BUN=Blood urea nitrogen

**Table-10:** Effects of black soldier fly larvae on plasma antioxidant capacity in laying hens.

Parameter	n	Fixed effect			Heterogeneity		
	
		SMD	95% CI	p-value	χ^2^	I^2^(%)	p-value
GSH-PX (U/mL)	264	1.39	1.12–1.66	<0.00001	12.94	92	0.0003
SOD (U/mL)	264	−2.38	−2.73–−2.02	<0.00001	106.59	99	<0.00001
MDA (nmol/mL)	264	4.78	4.29–5.27	<0.00001	17.99	94	<0.0001
CAT (U/mL)	264	−2.33	−2.75–−1.91	<0.00001	230.83	100	<0.00001

n=Total sample, SMD=Standard mean difference, 95% CI=95% confidence interval, I^2^=Primary index for reporting heterogeneity, GSH-PX=Glutathione peroxidase, SOD=Superoxide dismutase, MDA=Malondialdehyde, CAT=Catalase

## Discussion

In this meta-analysis, feeding BSFL meals had a significant effect on feed efficiency. These findings support previous studies reporting that BSFL supplementation at 8% [12], 5% [14], 17% [16], 12% [19], 10% [26], 75% [27], and 3% [28] can optimize feed efficiency. However, this alteration was unaffected by dietary interventions, feeding intake, or feed efficiency and cannot be attributed to the varying body weights of the hens. Low feed efficiency indicates that feed intake is appropriate [8]. Nutritional treatments administered to BSFLs did not affect feed intake or weight gain. Therefore, the feed may have impacted the increase in body weight. These findings suggest that hens can maintain their natural equilibrium and effectively utilize the protein included in the diet of BSFL without experiencing any metabolic disturbances [32].

Despite the high-fat content of BSFL, there was no direct correlation with body weight. Compared with broiler chickens, laying hens typically have the greatest impact of elevated fat levels in isoenergetic feed on egg yield. Increased levels of fat-dependent fatty acid linoleic acid and digestible energy may be connected to these effects [19]. This is because of the presence of chitin, which prevents digestion by binding to proteins. The inverse relationship between the amount of insect meal included in the diet was probably due to chitin’s ability to bind bile acids and reduce the amount of fat absorbed through the gastrointestinal tract. The chickens’ serum examination provides clear evidence of chitin’s positive impacts of chitin on poultry health and metabolism [33]. In comparison with the other groups, hens fed a larger quantity of insect-based meal exhibited higher globulin values and reduced albumen/globulin ratios [29].

Ensuring sufficient growth performance in chickens requires maintaining the gastrointestinal tract’s health and proper functioning. Nutrients and components of the diet may affect hen intestinal health [34]. To reduce mortality and maintain uniformity in juvenile laying hens, the starting phase is essential for maturation, bone ossification, and alimentary tract adaptation. This could have a direct impact on the subsequent performance of laying hens [35]. Full-fatted black soldier flies have balanced amino acid compositions and profiles of mono- and polyunsaturated fatty acids, making them superior to plant protein sources for feeding poultry [36]. Furthermore, several studies have demonstrated that adding full-fatted BSFL to the diet changed the free fatty acid profile, which in turn enhanced meat quality [37]. However, by lowering the amounts of plant-based negligible nutrients (i.e., non-starch polysaccharide), replacing the plant-derived protein of maize gluten-related and soybean meal with BSFL might improve digestible nutrients and clarify the advantages of BSFL consumption for production performance [27].

According to this study, the haugh unit was significantly affected by BSFL feeding, as shown in previous studies, at ratios of 7.5% [17], 12% [19], and 3% [20]. A treatment ratio of 8% also affected albumin levels [12]. An essential parameter for assessing internal egg properties is the haugh unit. A popular commercial indicator of albumen grade is the associated haugh unit, which represents the ratio of albumin height to egg weight. Albumen height is a crucial indicator of egg freshness and quality. It may be possible to produce eggs of higher albumin quality by regulating feed intake and weight gain [38]. In addition, a previous study discovered that eggs from layers fed on a time-restricted basis had significantly higher haugh unit grades than eggs from control chickens fed an ad libitum schedule [39]. In addition, previous studies by Heuel *et al*. [11], Navasero *et al*. [20], Park *et al*. [21], and Ukwu *et al*. [40] have demonstrated that chickens with lower body weights and feed consumption levels produce eggs with elevated albumen height and haugh units.

The ovomucin is responsible for the thick white viscosity, and the egg’s ovomucin content mostly affects the haugh unit measurements [41]. The components of an egg include the yolk (27.5%), albumen (63%), and eggshell (9.5%) [42]. Low dietary protein was found to reduce albumen synthesis, which in turn reduced albumen weight and solid content. Non-traditional plant proteins have been found to have a negative impact on albumen quality. This effect may be attributed to either an excess or a deficiency of specific amino acids, such as arginine, phenylalanine, histidine, and leucine, or to the existence of anti-nutritional components, such as phytic acid and tannin, which have been shown to affect the *in vivo* absorption of proteins and their deposition into albumin, particularly ovomucin [43].

According to several studies, laying hen diets could be improved with a combination of BSFL [13, 27], *Cirina forda* [44], *Locusta migratoria* [45], *Gryllodes sigillatus, Schistocerca gregaria*, and *Tenebrio molitor* [46] as potent alternatives for protein at various substitution ranges (using the aforementioned larvae >75% of the total), with no adverse effects on yolk weight. Conversely, a 50% substitute for *C. forda* meal greatly decreases albumin weight, whereas a complete replacement only slightly decreases albumin weight [47]. The findings verified that the egg component most vulnerable to novel protein sources is the albumen. The greater proportion of albumen in chicken eggs fed BSFL is the only reason for the decrease in yolk content [24]. It is unclear how insect food affects yolk color. A previous study found no effect or slight discoloration when 5% of BSFL meals were fed to the diet of an Arabian strain; however, the findings were not comparable due to the use of various color evaluation techniques. Furthermore, the carotene pigments and xanthophyll characteristics necessary for egg color are not found in insects [7].

According to the hematological analysis, there were no significant differences in the BSFL and control meals. This meta-analysis supports previous investigations by Irawan *et al*. [12] and Marono *et al*. [16], demonstrated that BSFL can replace soybean meal and basalt. The maintenance of physiological homeostasis is another important function of plasma proteins, and albumin is the most potent supply of amino acids required for protein synthesis [48]. Enhanced immune response and illness resistance in hens are indicated by a positive effect on albumin/globulin ratios [49]. In the meantime, chitin affects serum cholesterol and triglyceride levels. Chitosan, a chitin derivative, can bind lipids and stop the substances from being absorbed by completely blocking their emulsification in the intestine. Furthermore, chitin’s cytoprotective effect or its ability to inhibit cytochrome P450 may have contributed to its ability to lower blood bilirubin levels [50].

In this study, BSFL meals had beneficial effects on kidney, liver, and electrolyte physiology. In previous studies by Bovera *et al*. [10] and Marono *et al*. [16], BUN, uric acid, and creatinine levels were lower or nonsignificant in rats fed a basal diet. These findings revealed no significant effects on protein metabolism. The serum uric acid level indicates protein catabolism and is the most common nitrogenous waste product in hens [51]. Another crucial marker of protein metabolism is creatinine, which is produced when phosphocreatine is degraded in skeletal muscle. Blood creatinine levels correlate with age, muscle mass, physical activity level, and nutrition. Since creatinine is also regarded as an indicator of renal function and protein metabolism, the lowest amount of creatinine observed in hens-fed insect meal may be related to the lowest protein consumption in these animals [52]. We hypothesized that chitin contributes to the manner in which hens metabolize proteins, although there is no sufficient evidence in published research that chitin affects kidney function. However, a previous study by Anraku *et al*. [53] demonstrated that chitosan, which is synthesized commercially by deacetylating chitin, improves kidney function, as indicated by alleviation of creatinine levels in the blood.

Our meta-analysis investigation of antioxidant activity revealed interesting findings on the role of BSFL in GSH-PX and MDA levels, which are indicators of oxidative stress and lipid peroxidation. Superoxide dismutase and catalase levels were not substantially affected by the insect-based diet; however, MDA and GSH-PX levels indicated decreased oxidative stress and lipid peroxidation in the liver. Abnormal lipid metabolism is a potential problem associated with high MDA levels in the liver because it can impair metabolic activity [54]. Furthermore, unexpected elevations in serum AST, ALT, and ALP levels could indicate liver disorders. Consequently, the hepatoprotective effects of BSFL supplementation may be linked to more constant levels of ALT and ALP. This highlights the potential of BSFL as a food source to alleviate liver reactive oxygen species. An increase in oxidative enzyme expression in the liver of laying hens appears to be related to the presence of bioactive chemicals in insect-based meals [55]. Previous investigations by Bovera *et al*. [10] and Marono *et al*. [16] on the regulation of electrolytes similarly revealed alleviated levels of Mg, Cl, P, and Fe in BSFL-fed groups. One factor contributing to the maintenance of electrolyte levels in laying hen blood is the role of chitin as a sustainable and ubiquitous electrode binder for electrochemical capacitors [56]. However, humoral and neurological systems control mineral homeostasis [57]. The concentration of minerals in the feed and other factors affecting the degree of absorption in the digestive system significantly affects the mineral content of poultry serum [58].

The current meta-analysis demonstrates that feeding BSFL meals to laying hens enhances their feed efficiency, egg quality, and several physiological parameters. The broad range of outcomes revealed in the aforementioned studies provides context for the conclusions drawn from the earlier investigations. The dietary content of the BSFL meal that was employed can be affected by the insect’s life stage (adult, larva, or pupa), substrate used for insect rearing, defatting procedure, and growth season when the hens were fed may be connected to this heterogeneity. However, more investigation is required to fully comprehend the profitability of BSFL meals, particularly if changes are made to the formula’s content (g/100 kg) so that fish or soybean meal can be substituted. Prospective studies to determine the optimal BSFL diet formula for use with consistent and integrated parameter results should use this meta-analysis as a guide.

## Conclusion

A fixed-effects meta-analysis of 24 study findings, including 4,549 samples from 17 different countries on five continents, was used to investigate the effects of feeding BSFL meals on the production performance, egg quality, and physiological parameters of laying hens. The results of this study demonstrated that feeding laying hens BSFL meals had favorable effects on their feed efficiency, haugh unit, albumen quality, eggshell quality, serum glucose, lipid metabolism, renal and liver physiology, antioxidant activity, and electrolyte balance. Although the consumption of BSFL did not have a favorable impact on body weight gain, laying frequency, yolk quality, and hematological profile, this meta-analysis study highlighted that BSFL may be advantageous for the feed industry.

## Authors’ Contributions

MTEP and HC: Conceptualization, methodology, and data curation. AP, FF, and SC: Data extraction and editing. MTEP: Performed data analysis and edited the visualization and validation of tables and figures. MTEP and HC: Drafted and revised the manuscript. All authors have read, reviewed, and approved the final manuscript.
